# Food-Derived Bioactive Peptides on Inflammation and Oxidative Stress

**DOI:** 10.1155/2014/608979

**Published:** 2014-01-02

**Authors:** Subhadeep Chakrabarti, Forough Jahandideh, Jianping Wu

**Affiliations:** Department of Agricultural, Food & Nutritional Science, the Cardiovascular Research Centre, University of Alberta, 4-10 Ag/For Centre, Edmonton, AB, Canada T6G 2P5

## Abstract

Chronic diseases such as atherosclerosis and cancer are now the leading causes of morbidity and mortality worldwide. Inflammatory processes and oxidative stress underlie the pathogenesis of these pathological conditions. Bioactive peptides derived from food proteins have been evaluated for various beneficial effects, including anti-inflammatory and antioxidant properties. In this review, we summarize the roles of various food-derived bioactive peptides in inflammation and oxidative stress and discuss the potential benefits and limitations of using these compounds against the burden of chronic diseases.

## 1. Introduction

Chronic noncommunicable diseases such as cardiovascular diseases and cancer make up an increasing share of the global disease burden. With the increased longevity and improvement in global living standards, these “diseases of affluence” are now widespread in both developed and developing nations [1, 2]. Indeed, cardiovascular diseases such as atherosclerosis and its complications are now the leading cause of mortality and morbidity worldwide, closely followed by various cancers [3, 4]. Increased life spans have also meant corresponding increase in aging-related diseases in both developing and developed nations which may overwhelm their health care systems. Although atherosclerosis, cancers, and aging-related diseases can have diverse etiologies, they share many underlying pathological mechanisms including abnormalities in inflammatory responses and oxidative stress [5–7]. Thus targeting of the common pathological pathways has gained increasing attention in recent years for both prevention and treatment of chronic diseases.

While a number of commercially available anti-inflammatory and antioxidant drugs exist, none of these are free from side effects. Given the concerns about the side effects from prolonged usage of synthetic compounds, there is growing interest in the therapeutic applications of natural compounds and their derivatives as safer alternatives, either as functional foods or nutraceuticals. Food proteins from both plant and animal sources have been used to obtain a wide range of bioactive peptides [8]. Bioactive peptides are generally short peptides (3–20 amino acids) derived from proteins that can exert biological activities over and above their expected nutritional value [9]. These peptides are often functionally inactive within the native proteins and must be released by proteolysis (*in vivo* digestion, *in vitro* enzymatic hydrolysis, or bacterial fermentation) to achieve their specific “bioactive” roles. Many of these food-derived peptides demonstrate antihypertensive, anti-inflammatory, antidiabetic, and antioxidant properties under experimental conditions [10–12]. While some studies have observed the effects of single peptides, many others have examined protein hydrolysates composed of a mixture of diverse bioactive peptides [13–15]. Given their food-based sources and a perceived lack of serious side effects, bioactive peptides and peptide-rich protein hydrolysates can potentially provide a better alternative to synthetic pharmaceuticals for the prevention and treatment of chronic illnesses that affect an increasing number of people.

While bioactive peptides and peptide-rich protein hydrolysates can have a range of beneficial effects on diverse pathological conditions, this review would mainly focus on their anti-inflammatory and antioxidant actions. We would also discuss the potential challenges that may limit the use of these compounds as novel therapies against the global burden of chronic diseases.

## 2. Bioactive Peptides on Inflammation

### 2.1. Inflammation and Chronic Disease

Inflammation is the body's response to nonlethal injury which is characterized by increased endothelial permeability, leakage of protein-rich exudates, and infiltration of leukocytes into extravascular tissues. While inflammation is essential for resistance to microbial infections and wound healing, excessive and uncontrolled inflammatory changes often lead to chronic diseases. Indeed, vascular inflammation is an early event in the development of atherosclerosis and its complications such as myocardial infarction and stroke. Increasing evidence also links chronic inflammation to many types of cancer which further highlights its key role as a mediator of non-communicable illnesses. Despite the significance of inflammation, relatively few therapies have been devised to target the inflammatory component of cardiovascular and malignant diseases. The nonsteroidal anti-inflammatory drugs (NSAIDs) like aspirin are widely used to prevent and manage cardiovascular diseases, due to its antithrombotic as well as anti-inflammatory properties [16, 17]. Recent studies suggest that NSAIDs may also contribute to beneficial effects against cancers of the gastrointestinal system, further broadening the potential for anti-inflammatory therapies [18]. However, the presence of well-known side effects such as gastric bleeding and ulceration preclude the long-term use of NSAIDs for a large part of the population.

### 2.2. Pathways of Inflammatory Response

Inflammation is a complex and multisystem event affecting a wide range of cells, tissues, and organs. The vascular endothelium plays a key role as a gate keeper for the extravasation of leukocytes which is a hallmark of inflammation. However, tissue macrophages, epithelial cells, and fibroblasts are often involved in the generation of mediators which impinge upon and subsequently activate the endothelium through expression of leukocyte adhesion molecules like intercellular adhesion molecule-1 (ICAM-1) and vascular cell adhesion molecule-1 (VCAM-1) which recruit leukocytes from the bloodstream and lead to their extravasation through a sequential cascade that involves tethering, rolling, activation, firm adhesion, and, finally, transmigration across the endothelial barrier. Mediators like various proinflammatory cytokines (such as tumor necrosis factor and interleukin (IL)-1*β*), chemokines (such as IL-8 and monocyte chemoattractant protein-1 (MCP-1)), and reactive oxygen species (ROS, such as superoxide and peroxynitrite) are involved in both the generation and propagation of the inflammatory response. A number of intracellular signaling pathways are activated in the participating cells which include nuclear factor-kappaB (NF-*κ*B), mitogen activated protein (MAP) kinases, and activator protein-1 (AP-1), to name a few [19, 20]. Thus, the markers of inflammation include activation of proinflammatory signaling cascades, upregulation of leukocyte adhesion molecules, tissue infiltration of leukocytes, and increased levels of cytokines and chemokines in the circulation. Given the complexity and diversity of the inflammatory response, an investigation of potential anti-inflammatory agents involves the study of their effects on several of these markers, often using different cellular and intact animal systems for validation.

### 2.3. Bioactive Peptides on Inflammation in Cellular Systems

Much of the recent knowledge on bioactive peptides has been based on studies performed in cultured mammalian cells. Cell culture systems offer fast, economically feasible, and reproducible assays to analyze and validate the effects of many different compounds on a wide range of inflammatory markers. Peptides and protein hydrolysates derived from food sources such as milk, egg, fish, meat, and soybeans (to name a few) have all been tested for potential beneficial effects in these systems.

Bioactive peptides from milk have been among the first food-derived peptides studied. Milk is rich in caseins and whey proteins, both of which can give rise to a number of peptides with bioactive properties upon further processing such as enzymatic hydrolysis, digestion, and/or fermentation. The tripeptides VPP and IPP, derived from bacterial fermentation of casein, demonstrate inhibitory effects on angiotensin converting enzyme (ACE) in addition to stimulation of nitric oxide (NO) and bradykinin-mediated vasorelaxant pathways, thus suppressing the prohypertensive and proinflammatory mechanisms associated with hypertension and atherosclerosis [21]. Recently, a more direct anti-inflammatory role for VPP has been shown by its ability to attenuate leukocyte-endothelial interactions *in vitro*, largely through inhibition of proinflammatory c-Jun N-terminal kinase (JNK, a type of MAP kinase) pathway [22]. Casein hydrolysates generated by enzymatic digestion and containing a mixture of peptides have also been evaluated for anti-inflammatory properties. For example, digestion of casein with Corolase yields preparations that demonstrate anti-inflammatory effects on activated macrophages [23]. Hydrolysates of whey proteins also show promise in inhibition of inflammatory responses in respiratory and intestinal epithelial cells [24, 25]. Lactoferrin is a milk protein with antimicrobial properties which also exerts anti-inflammatory effects on activated macrophages [26]. Hydrolysis of lactoferrin yields the bioactive peptide lactoferricin, which demonstrates anti-inflammatory effects on human cartilage and synovial cells, suggesting potential benefits in arthritis management [27, 28]. In addition, both human and animal milk contain a number of anti-inflammatory and immunomodulatory compounds such as transforming growth factor-beta (TGF-beta), IL-10, and immunoglobulins which can further modulate the immune system of the gastrointestinal tract; however, these are not strictly “bioactive” peptides as they do not require processing from the native protein for their actions (reviewed in [11, 29, 30]).

Egg is another nutritious dietary component that is a source for many bioactive peptides [31]. Work from our lab has demonstrated the generation of egg tripeptides (IRW and IQW) from ovotransferrin (an egg white component) which are effective in the downregulation of cytokine-induced inflammatory protein expression in vascular endothelium, at least partly through the modulation of NF-**κ**B pathway [32, 33]. These anti-inflammatory properties are also observed in conjunction with antioxidant and ACE inhibitory effects, further enhancing the beneficial actions [34]. Interestingly, these beneficial effects require the presence of an intact tripeptide as the corresponding dipeptides and constituent amino acids alone failed to replicate the anti-inflammatory functions, indicating a structure-function relationship between the tripeptide structure and blockade of inflammation [32].

Fish and meat are important sources of dietary protein. Recent findings suggest they also contribute to human health through generation of bioactive peptides; however, detailed studies at the cellular and molecular level are still quite sparse. A fish hydrolysate preparation has been shown to induce proliferation and migration in intestinal epithelial cells, which may contribute to anti-inflammatory and healing properties [13].

Plant-derived foods are another important source for bioactive compounds including many peptides and protein hydrolysates. Soybean hydrolysates have yielded several bioactive peptides with anti-inflammatory effects on macrophage cell lines, with preparations from germinated beans eliciting the stronger responses [35]. Chungkookjang, a fermented soybean product from Korea, is rich in bioactive peptides and shows anti-inflammatory effects in breast cancer cells by downregulation of cytokine/chemokines expression and activation of transforming growth factor (TGF)-beta signaling [36]. One of the soybean-derived peptides, lunasin, appears to exert widespread anti-inflammatory effects including suppression of NF-**κ**B activity, reduced cytokine expression, and reduction in cyclooxygenase-2 (COX-2) levels in addition to its antioxidant and anticarcinogenic properties [37, 38]. The presence of an RGD motif in lunasin and similar peptides is believed to contribute to their anti-inflammatory effects, potentially involving antagonism of integrin signaling and downstream proinflammatory cascades [39].

### 2.4. Bioactive Peptides on Inflammation *In Vivo*

Based on the encouraging findings from cell-based studies, several bioactive peptides and hydrolysates have now been tested in animal models of human diseases. A number of different inflammatory models, typically experimentally induced colitis, arthritis, atherosclerosis, and respiratory tract inflammation, have been used. As much of this work has been performed only within the last few years, large-scale human trials are still lacking, although a few smaller studies on humans have shown some therapeutic promise.

Not surprisingly, milk-derived peptides have been in the forefront of *in vivo* studies of anti-inflammatory properties. The tripeptides VPP and IPP appear to be beneficial in a model of intestinal enterocolitis by their mediation of anti-inflammatory effects [40]. In addition, these peptides offer protection against the development of atherosclerotic changes in the apolipoprotein E (ApoE) knockout mice through a concerted action that involves modulation of both inflammatory and hypertensive pathways [41]. Hydrolysates of whey proteins can attenuate the dermatitis in NC/Nga mice [42], while casein hydrolysates (such as those produced by *Aspergillus oryzae* protease or fermentation with thermophilic lactobacilli) have shown promise in treating adjuvant arthritis in rats and chemically induced colitis in mice through modulation of both chronic and acute inflammatory responses [43, 44].

Other peptides and protein hydrolysates from animal sources have been used in several animal models of disease. In our lab, the egg-derived tripeptide IRW has shown promise in controlling both the hyperactive renin-angiotensin system (RAS) pathway as well as the exaggerated proinflammatory phenotype in spontaneously hypertensive rats (SHRs), a widely used model of hypertension and cardiovascular disease [45]. Fish protein hydrolysates have demonstrated protective effects on different murine models of colitis, including those induced by dextran sulphate as well as by chronic NSAID usage, suggesting their potential applications in human disease [46–48]. A similar preparation also reduced markers of inflammation and improved the plasma lipid profile in high fat-fed mice, with potential implications for obesity-induced inflammation and vascular disease [49]. Chicken collagen hydrolysate (CCH) containing an array of bioactive peptides has been used in rodent models of cardiovascular diseases. In the ApoE deficient mice, CCH administration successfully reduced the plasma levels of inflammatory cytokines in addition to improving the plasma lipid profile [50]. CCH given to SHRs reduced blood pressure and circulating inflammatory markers while increasing the bioavailability of the beneficial vasorelaxant NO [51]. A pilot study on human volunteers has also confirmed the antihypertensive effects of CCH although the potential anti-inflammatory mechanisms, if any, remain to be determined [52].

A number of plant-derived bioactive peptides and peptide-rich hydrolysates have also been tested by *in vivo* studies. Feeding of soy protein isolate to rodents (which presumably generates bioactive short peptides through intestinal digestion) has shown beneficial effects on experimentally induced arthritis [53] and genetically predisposed atherosclerosis [54], through the induction of protective anti-inflammatory effects. Soybean peptides such as VPY and others have shown promise in controlling cytokine/chemokines levels, reduction of oxidative stress, and reversal of the tissue damage observed in animal models of colitis, suggesting potential applications in treatment of inflammatory bowel diseases [55, 56]. Oral intake of a corn gluten hydrolysate also reduced inflammatory injury in a rat model of experimental colitis [57]. Similarly, ingestion of pyro-glutamyl leucine (a bioactive peptide from wheat gluten hydrolysate) was shown to protect against dextran sulphate-induced colitis in mice [58] and chemically induced hepatitis in rats [14], further supporting the *in vivo* anti-inflammatory functions of plant-derived peptides.

**Figure 1:**
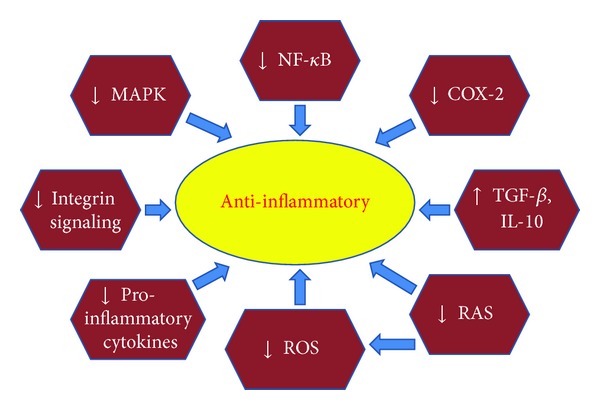
The potential mechanisms of action of anti-inflammatory bioactive peptides and peptide-rich protein hydrolysates. MAPK: mitogen activated protein kinase; NF-**κ**B: nuclear factor-**κ**B; COX-2: cyclo-oxygenase-2; TGF-beta: transforming growth factor-beta; IL-10: interleukin-10; RAS: renin-angiotensin system; ROS: reactive oxygen species.

These anti-inflammatory effects of bioactive peptides and hydrolysates have been summarized in Table 1. A schematic diagram of the potential anti-inflammatory mechanisms of bioactive peptides is also shown (Figure 1) demonstrating the effects of these compounds on proinflammatory signaling kinases, pro- and anti-inflammatory cytokines, integrin-dependent signaling, ROS generation, and the renin-angiotensin system.

## 3. Bioactive Peptides on Oxidative Stress

### 3.1. Oxidative Stress and Chronic Disease

The term ROS encompasses a range of oxygen-containing highly reactive species including free radicals superoxide (O_2_^−•^) and hydroxyl radicals (HO^•^) as well as nonradical form like hydrogen peroxide (H_2_O_2_), hypochlorous acid (HOCl), singlet oxygen, and peroxynitrite (ONOO^−^) [59]. In low concentrations, ROS can be actually beneficial by induction of apoptosis in damaged/aged cells, detoxification of xenobiotics by cytochrome P450 system, and killing invading microorganisms by phagocytes and as regulatory mediators in cell signaling pathways [60, 61]. However, an excess of ROS, both due to excessive production or impaired antioxidant capacities or both, is harmful and leads to what is known as oxidative stress.

In pathological conditions, ROS attack nucleic acids (DNA or RNA), proteins, and unsaturated fatty acids and aggravate cellular damage (reviewed in [62, 63]). One example of DNA lesions is the conversion of guanine to 8-hydroxyguanine which affects the methylation of cytosine. Normal methylation of cytosine is considered as a critical step in regulation of gene expression and once it is altered, it may contribute to carcinogenesis [64]. Apart from DNA, peroxyl radicals (ROO^•^) can also initiate peroxidation of fatty acids. The final products of this reaction are malondialdehydes (MDA) which possess carcinogenic properties [65]. Proteins are another group of macromolecules affected by the ROS. Cleavage of the peptide bond, amino acid modification, and formation of cross-linked peptide aggregates happen during protein oxidation by ROS that leads to formation of protein derivatives possessing highly reactive carbonyl groups (ketones and aldehydes) which are involved in the complications of diabetes and many age-related diseases [66].

In addition to destructive effects on macromolecules, ROS also impair vasodilatory responses by reaction with NO. The reaction between NO and O_2_^−^ results in the production of peroxynitrite (ONOO^−^), which reduces the bioavailability of NO which is a potent vasorelaxant signaling messenger in vascular system. Increased oxidative stress and its downstream effects can lead to various conditions such as cardiovascular diseases [67], Alzheimer's disease [68], aging [69], and cancer [70]. Dietary intake of antioxidant compounds can reinforce the body's oxidant status and help to maintain a balanced condition in terms of oxidant/antioxidant in the body. Given this background, there is increasing interest in food proteins and their constituent peptides as potential candidates for use as antioxidants.

### 3.2. Bioactive Peptides as Antioxidants: Cell-Free Systems

Several chemical methods with different mechanisms of action have been developed to measure antioxidant potential of food proteins and peptides. This is because of complexity of oxidative reactions taking place in biological systems. Scavenging of stable free radicals 2,2-diphenyl-1-picrylhydrazyl (DPPH) and 2,2′-azino-bis(3-ethylbenzothiazoline-6-sulphonic acid (ABTS) methods), reduction of metal ions (ferric ion reducing antioxidant power (FRAP) and cupric ion reducing antioxidant capacity (CUPRAC) methods), protecting a target molecule by inhibition of its consumption (oxygen radical absorbance capacity (ORAC) and total radical-trapping antioxidant potential (TRAP) assays), and inhibition of low density lipoprotein (LDL) oxidation are some of the common strategies used in chemical-based antioxidant assays (reviewed in [71, 72]). While the DPPH-based assay was among the first ones to be used extensively [73–76], many such assays have been widely used for screening antioxidant peptides.

A wide range of antioxidant peptides have been identified from marine organisms including oyster, shrimp, squid, blue mussel, and a variety of fish species (tuna, sardine, hoki, sole, and pacific hake) after hydrolysis with different enzymes. Puffer fish hydrolysate produced strong antioxidant action as shown by the ORAC assay compared to many other fish sources [77]. Both the salmon protein hydrolysate and peptide fractions inhibited the oxidation of linoleic acid [78]. Flounder fish muscle hydrolyzed with *α*-chymotrypsin has been also reported to possess strong antioxidant activities by scavenging free radicals *in vitro* [79]. Blue mussel (*Mytilus edulis*) is another source for the production of antioxidant peptides. Hydrolysis of this protein by the enzyme neutrase could scavenge 30% of DPPH radicals while further purification of this hydrolysate revealed the active peptide with the sequence of YPPAK with enhanced hydroxyl and superoxide anion radical scavenging activities [80].

Milk proteins also contribute much in the context of antioxidant peptides. YFYPEL, a hexapeptide isolated from pepsin hydrolysate of bovine casein, showed antioxidant activity by scavenging superoxide, DPPH, and hydroxyl radicals *in vitro* [81]. In a recent study, whey protein concentrates (WPC) hydrolyzed by Corolase or thermolysin were investigated for antioxidant activity by ORAC assay. Thermolysin-hydrolyzed WPC (8 hrs at 80°C, enzyme/substrate ratio: 0.10 w/w) was the most potent hydrolysate with radical scavenging activity and several peptides were identified in this hydrolysate [82]. Ovine **κ**-casein antioxidant activity also increased by 3-fold upon hydrolysis with pepsin, trypsin, and chymotrypsin. This casein hydrolysate further inhibited lipid peroxidation and several peptides contributing to antioxidant activity were identified [83]. Goat milk casein also exhibited enhanced free radical scavenging and metal ion chelating activity following hydrolysis by a combination of neutral and alkaline proteases. Further purification revealed five novel peptides in this hydrolysate with potential antioxidant properties [84].

Plants are known for antioxidant effects mostly because of their polyphenolic compounds. However recent research indicates the significance of many plant proteins and peptides as novel antioxidant agents. The potential of commercially available microbial proteases to enhance antioxidant potential of soy and corn proteins has been recently demonstrated [85, 86]. Corn protein hydrolysates were more effective than soy protein hydrolysate and inhibited lipid oxidation by 53% at lower incorporated dosage (200 *μ*g/g) while soy protein hydrolysate reduced oxidation by 20% at much higher dosage (800 *μ*g/g) [85]. In a recent study the antioxidant activity of chickpea albumin hydrolysate through *in vitro* radical scavenging and reducing power assays has been assayed. Further purification of the hydrolysate fraction with highest antioxidant activity identified RQSHFANAQP as the active component responsible [87]. All of the aforementioned peptides were evaluated for antioxidant activity through *in vitro* methods. Although these methods are good for screening and assessing preliminary data, there are drawbacks associated with these chemical-based assays including potential lack of relevance to biological systems and altered mechanisms of free radical generation [72, 88]. Therefore, it is preferable to use at least two different chemical assays prior to validation of antioxidant activity in more physiologically relevant systems like cells and whole organisms.

### 3.3. Bioactive Peptides as Antioxidants: Cellular Systems

Cell-based assays as intermediate methods have been used increasingly recently to evaluate the protective effects of antioxidants against oxidative stressors and to elucidate mechanism of action of peptides within cells [89]. Cell-based methods can be used to elucidate the mechanism/s of action of antioxidant agents within live cells. Moreover, cell culture assays are useful for the determination of peptide dosage to exert beneficial antioxidant effects without cytotoxicity for *in vivo* experiments. Among animal source peptides, those from flounder fish protein hydrolysates showed antioxidant and cytoprotective effects against 2,2′-azobis-(2-amidinopropane) dihydrochloride (AAPH) without cytotoxicity in the range of 12.5 to 200 *μ*g/mL in Vero cells, a monkey kidney fibroblast line [79]. An antioxidant peptide from skin gelatin hydrolysate of hoki fish increased expression of cellular antioxidant enzymes including catalase (CAT), superoxide dismutase (SOD), and glutathione peroxidase (GPx) in human hepatoma cells (Hep3B) [90].

A recent study on bacterial fermentation in sourdough showed antioxidant effects on cultured fibroblasts [91]. Hempseed-derived antioxidant peptides also exhibited protective effects against oxidative apoptosis in rat pheochromocytoma line PC12 cells [92]. Similar effects were exerted by a hydrolysate of rice endosperm protein on mouse macrophages [93]. Results of these cell-based assays clearly show antioxidant potential of food peptides beyond the free radical scavenging in chemical assays.

### 3.4. Bioactive Peptides as Antioxidants: *In Vivo* Effects

*In vivo* studies such as animal experiments or human trials should be conducted after identification of an antioxidant peptide through *in vitro* and cell-based assays to demonstrate its antioxidant activity in an intact organism. Despite the importance of *in vivo* studies for verifying the antioxidant activity of bioactive peptides, only a few studies on the efficacy of antioxidant peptides have been conducted in animal models. Long-term administration (17 weeks) of egg white hydrolysate to SHRs has been shown to improve the plasma antioxidant capacity. Moreover MDA levels decreased significantly in the aortic tissue of rats receiving 0.5 g/kg/day and reverted to baseline levels five weeks after the treatment [94]. Similarly, recent studies on whey protein consumption also suggest a number of benefits including reduction of oxidative stress and better management of metabolic syndrome in both animal models and human subjects (reviewed in [12]). Among plant-derived sources, rapeseed crude protein hydrolyzed with Alcalase and Flavourzyme has shown to be a potential source for antioxidant peptides. Rapeseed hydrolysates were intraperitoneally injected to rats at 50 or 100 mg/kg/day for 16 days. At the end of the experiment, the serum was collected when 12.8% and 46.9% reduction in MDA levels were observed for 50 and 100 mg/kg/day dosages of rapeseed protein hydrolysate, respectively [95]. The findings from various cell-based and *in vivo* studies have been summarized in Table 2.

## 4. Potential Challenges and Opportunities

### 4.1. Limitations and Risks

While the field of bioactive peptides is an exciting and growing area of research, there are a few risks and limitations before the widespread use of such peptides in the general population. As previously discussed, many of the studies are still at an early stage and more *in vivo* data will be needed before applications to human health. In the absence of solid pharmacokinetic data, proper dosage and frequency of administration may be impossible to determine, leading to wide variability in intake and biological effects [29, 96]. While these bioactive peptides are considered to be relatively safe, there is always the risk of potential side effects if too high a dose is consumed. For example, antioxidant vitamins were traditionally considered safe even in high doses; yet recent evidence suggests potential toxic effects on excessive consumption [97, 98]. Another potential risk could be due to the presence of immunogenic proteins and peptides within the protein hydrolysates, which may induce and/or exacerbate allergic reactions in a minority of users [99, 100]. Proper screening prior to the ingestion of such hydrolysates might be necessary in subjects prone to allergies. While this lack of knowledge about specific compounds and their potential side effects is a limitation, it also provides opportunities for future research in several directions.

### 4.2. Future Directions

Future studies in the field of bioactive peptides would likely involve detailed studies on animals and human volunteers to better understand the pharmacokinetics of these compounds, testing for potential immunogenicity (to prevent allergies), characterizing individual components of complex peptide-rich hydrolysates to tease out their specific actions as well as basic biomedical research directed towards identifying specific receptors and signaling pathways involved in mediating some of these beneficial anti-inflammatory and antioxidant actions. Indeed, outside the renin-angiotensin system [101, 102], few receptors have been identified as involved in bioactive peptide actions. Further work on identifying specific peptide sequences and their corresponding receptors may provide opportunities for better targeting of inflammation and oxidative stress in a tissue- and organ-specific manner.

## 5. Conclusions

Bioactive peptides and peptide-rich protein hydrolysates represent a new direction in functional foods and nutraceuticals. While both types of preparations have shown promise as potential anti-inflammatory and antioxidant agents, further research is still needed to verify these beneficial effects in order to successfully translate the research from bench to the bedside to effectively control the growing burden of chronic noncommunicable illnesses with minimal side effects.

## Figures and Tables

**Table 1:** Food-derived anti-inflammatory peptides/hydrolysates in cell-based and *in vivo* systems.

Protein source	Preparation	Active component	Cell/organism tested in	Observed effects	Reference
Casein	Bacterial fermentation	VPP	Endothelial cells and leukocytes	Reduced leukocyte recruitment	[22]
		VPP, IPP	Murine colitis	Anti-inflammatory	[40]
Casein	Corolase hydrolysis	Hydrolysate	Macrophages	Downregulation of COX-2, NF-*κ*B inhibition	[23]
	*Aspergillus oryzae* protease hydrolysis	Hydrolysate	Rat adjuvant arthritis	Reduced arthritic score, anti-inflammatory	[43]
Whey protein	Enzymatic hydrolysis	Hydrolysate	Epithelial cells	Reduced cytokine expression	[24, 25]
			NC/Nga mouse	Reduced dermatitis	[42]
Lactoferrin	Enzymatic hydrolysis	Lactoferricin	Synovial cells	Anti-inflammatory, antiarthritis	[27, 28]
Ovotransferrin	Thermolysin and pepsin hydrolysis	IRW, IQW	Human endothelial cells	Reduced ICAM-1/VCAM-1 with cytokine treatment	[32]
Fish protein	Enzymatic hydrolysis	Hydrolysate	Intestinal epithelial cells (human and rat)	Anti-inflammatory, increased proliferation	[13]
			Murine colitis (DSS/NSAID induced)	Reduced cytokines, improved healing	[46]
			High fat-fed mouse	Improved lipid profile	[49]
Chicken collagen	Acid treatment followed by *Aspergillus oryzae* protease hydrolysis	Hydrolysate	ApoE knockout mouse	Reduced cytokines, improved plasma lipid profile	[50]
			SHR (rat)	Reduced ICAM-1 and decreased blood pressure	[51]
Soy protein	Fermentation	Chungkookjang	Breast cancer cells	Anti-inflammatory, increased TGF-beta	[36]
Soy protein	Enzymatic hydrolysis	Lunasin	Macrophage	Reduced cytokines, NF-*κ*B inhibition	[37]
		VPY	Murine colitis	Reduced cytokines, reduced oxidative stress, and improved histology	[55]
Wheat gluten	*Aspergillus oryzae* protease hydrolysis and fractionation	Pyro-glutamyl leucine	Rat hepatitis	Anti-inflammatory, improved hepatic enzyme profile	[14]
			Mouse colitis	Improved mucosal histology and less weight loss	[58]

**Table 2:** Food-derived antioxidant peptides/hydrolysates in cell-based and *in vivo* systems.

Protein source	Preparation	Active component	Cell/organism tested in	Observed effects	Reference
Ovotransferrin	Thermolysin and pepsin hydrolysis	IRW, IQW	Endothelial cells (human)	Reduced cellular superoxide (dihydroethidium staining)	[32]
Chicken egg white	Hydrolysis with pepsin	Egg white hydrolysate	SHR (rat)	Increase in plasma radical scavenging, reduction in aorta MDA levels	[94]
Hoki skin gelatin	Hydrolysis with trypsin	HGPLGPL	Hep3B (human hepatoma cells)	Increase in cellular antioxidant enzymes (catalase, SOD, GPx)	[90]
Flounder fish protein	Hydrolysis with *α*-chymotrypsin	CAAP, VCSV	Vero cells (monkey kidney fibroblast cell line)	Cytotoxic protective effects, scavenging intracellular ROS	[79]
Cereal flours	Fermentation of sourdough with lactic acid bacteria	25 peptides (8–57 amino acid residues)	Mouse fibroblasts (Balb 3T3)	Protective effects against oxidative stress in fibroblasts	[91]
Hempseed	Alcalase hydrolysate of hempseed protein isolate	NHAV HVRETALV	Rat pheochromocytoma line PC12 cells	Protective effects against cell death/oxidative apoptosis	[92]
Rice endosperm protein	Neutrase hydrolysate of defatted rice endosperm protein	FRDEHKK	Mouse macrophage (RAW 264.7)	Scavenging of intracellular ROS (DCFH-DA method)	[93]
Rapeseed	Hydrolysis with Alcalase and Flavourzyme	Rapeseed crude hydrolysate	Wistar rat	50% reduction in serum MDA levels	[95]
